# Effect of Vitamin D Status during Pregnancy on Infant Neurodevelopment: The ECLIPSES Study

**DOI:** 10.3390/nu12103196

**Published:** 2020-10-19

**Authors:** Núria Voltas, Josefa Canals, Carmen Hernández-Martínez, Núria Serrat, Josep Basora, Victoria Arija

**Affiliations:** 1 Research Group in Nutrition and Mental Health (NUTRISAM), Universitat Rovira i Virgili, 43204 Reus, Spain; nuria.voltas@urv.cat (N.V.); josefa.canals@urv.cat (J.C.); carmen.hernandez@urv.cat (C.H.-M.); 2 Research Center for Behavior Assessment (CRAMC), Universitat Rovira i Virgili, 43002 Tarragona, Spain; 3 Institut d’Investigació Sanitària Pere Virgili (IISPV), 43007 Reus, Spain; 4 Laboratory ICS Tarragona, University Hospital of Tarragona Joan XXIII, Catalan Institute of Health (ICS), 43005 Tarragona, Spain; nserrat.tgn.ics@gencat.cat; 5 Collaborative Research Group on Lifestyles, Nutrition and Smoking (CENIT), Jordi Gol Primary Care Research Institute (IDIAPJGol), Camí de Riudoms 53-55, 43202 Reus, Spain; josep.basora@urv.cat; 6 Jordi Gol Primary Care Research Institute (IDIAPJGol), Camí de Riudoms 53-55, 43202 Reus, Spain

**Keywords:** vitamin D, pregnancy, prenatal nutrition, neurodevelopment, infant

## Abstract

Vitamin D status during pregnancy is involved in numerous physiological processes, including brain development. In this study, we assess the association between vitamin D status during pregnancy and infant neurodevelopment (cognitive, language, and motor skills). From an initial sample of 793 women (mean age 30.6) recruited before the 12th week of pregnancy, 422 mother–infant pairs were followed up to a postpartum visit. Vitamin D levels were assessed in the first and third trimesters of pregnancy, and socio-demographic, nutritional, and psychological variables were collected. At 40 days postpartum, the Bayley Scales of Infant Development-III were administered to the infants and several obstetrical data were recorded. Independently from several confounding factors, deficient vitamin D levels in the first trimester of pregnancy (<30 nmol/L) predicted a worse performance in cognitive and language skills. Language performance worsened with lower vitamin D levels (<20 nmol/L). In the third trimester, this highly deficient level was also associated with lower motor skills. Vitamin D deficiency was therefore associated with worse neurodevelopmental outcomes. More studies are needed to determine specific recommendations with regard to vitamin D supplementation during pregnancy in order to promote an optimal course for pregnancy and optimal infant neurodevelopment.

## 1. Introduction

Vitamin D is necessary for numerous physiological functions and biological processes. Adequate levels of vitamin D in the prenatal period are essential for the correct course of pregnancy and child development. The high prevalence of hypovitaminosis D worldwide has therefore become a major public health problem that affects all age groups. One of the main risk groups is pregnant women [1]. A review conducted by Palacios et al. [2] showed that the prevalence of vitamin D insufficiency, (defined as <75 nmol/L) in pregnant and lactating women, was around 33% in the United States, 20–70% in European countries, 67–96% in Asian countries, and 48% in Australia. Similarly, the prevalence of vitamin D deficiency (defined as <50 nmol/L) was 4–23% in European countries, 38–60% in Asian countries, around 15% in Australia and 48% in the United States [2,3]. A review by Karras et al. [4] on hypovitaminosis D in pregnant women from Mediterranean countries found that the prevalence of insufficiency and vitamin D deficiency was 9.3–41.4% and 22.7–90.3%, respectively. Several variables may be related to the differences in prevalence rates across studies. These include study design, the moment in pregnancy at which vitamin D is assessed, the season, supplementation, and the participants’ geographical area or ethnicity. A lack of consensus also exists on the criteria for normal, insufficient and deficient levels of vitamin D and on the effects deficient levels may have [5].

Interest in studies of hypovitaminosis D during pregnancy and its consequences on maternal and infant health has been growing recently. Deficiency could be associated with poor health outcomes during pregnancy, such as preeclampsia, gestational diabetes mellitus, bacterial vaginosis, or foetal infections [6,7]. For infants, a lack of vitamin D during pregnancy has been associated with prematurity, low birth weight, cardiovascular disease risk factors, abnormal skeletal development, asthma, and neurocognitive development problems [7,8,9]. Vitamin D therefore appears to play an important role in several child neurodevelopment issues and to have certain neuroprotective factors. However, few studies have focused on these topics and their findings are not conclusive [10,11,12]. Two recent systematic reviews concluded that low prenatal vitamin D status was associated with adverse infant neurodevelopment in cognitive, language, and motor skills [5,13] (Janbek, Specht, and Heitmann, 2019; Villalobos, Tous, Canals, and Arija, 2019). Darling et al. [14] suggested that, as well as motor skills, deficient prenatal vitamin D status may also have adverse effects on certain social development measures in children under four. Similarly, a metanalysis of 25 epidemiological studies supported evidence that high 25-hydroxyvitamin D3 (25(OH)D3) levels (a pheromone produced in the liver by the hydroxylation of vitamin D3 (cholecalciferol)) were associated with improved cognitive development and lower risks of attention deficit hyperactivity disorder and autism-related traits later in life [15]. In a Spanish population-based cohort study (INMA Project) conducted with 1820 mother–infant pairs, infants of mothers with higher circulating concentrations of 25(OH)D3 between the end of the first trimester and the beginning of the second trimester of pregnancy (>75 nmol/L) showed higher mental and psychomotor scores than those of mothers with lower concentrations (<50 nmol/L) [12]. In line with the above research, Zou et al. [16] recently observed a relationship between vitamin D in pregnancy and brain morphology in offspring. In a sample of 2597 preadolescents, the authors found that a higher maternal 25(OH)D3 concentration in mid-gestation was related to a larger cerebellar volume. Moreover, children exposed to a steadily deficient 25(OH)D3 concentration between this moment of pregnancy and delivery showed lower volumes of cerebral grey matter and white matter.

As the above studies have shown, vitamin D is involved in children’s health and neurodevelopment. However, research into the effects of prenatal hypovitaminosis D on neurodevelopment is still limited. The main aim of the present study is to investigate whether circulating 25(OH)D3 concentration at two stages of pregnancy (first and third trimesters) is associated with early neurodevelopmental outcomes in a Spanish sample of mother–infant pairs while taking into account several confounding variables. We tested whether vitamin D levels classified as highly deficient, deficient, insufficient, and normal have significant consequences on neurodevelopment. As suggested by previous studies, we hypothesize that deficient levels of vitamin D in the first and third trimesters of pregnancy are related to significantly worse scores on neurodevelopmental indices.

## 2. Materials and Methods

### 2.1. Study Design and Procedure

The ECLIPSES study [17] was a community randomized controlled trial (RCT) conducted in the province of Tarragona (Catalonia, Spain) between 2013 and 2017. The 793 participants (mean age 30.6 ± 5.1 years) were recruited by midwives at their primary care centres during their first pregnancy visit. The women were included in the trial in accordance with the following inclusion criteria: age 18 years or above, ≤12 weeks of gestation, absence of anaemia (Hb > 110 g/L at 12th week), and ability to understand an official language of the state (Spanish or Catalan) as well as the study characteristics. Excluded from the study were women with multiple pregnancies or adverse obstetric history, who had taken >10 mg of iron every day in the three months prior to the 12th week of gestation, those who reported prior severe illness (state of immunosuppression) or chronic disease that could affect their nutritional status (cancer, diabetes, etc.), and those who reported liver disease.

In addition to the recruitment visit before the 12th week of gestation, the study consisted of three other visits during pregnancy (in the 12th, 24th, and 36th weeks) and a final visit 40 days after delivery (postpartum visit). The women also attended routine visits with their midwives and gynaecologists during pregnancy, in which they could express their doubts and concerns and the clinical staff recorded any problems that arose. In total, 422 mother–infant pairs had their vitamin D levels assessed in the 1st and 3rd trimesters and the children’s neurodevelopment was assessed at the postpartum visit.

The study was designed in accordance with the Declarations of Helsinki and the Tokyo update. All procedures involving human subjects were approved by the Clinical Research Ethics Committee of the Jordi Gol Research Institute in Primary Care (IDIAP), the Pere Virgili Health Research Institute (IISPV) (155/2017), and the Spanish Agency of Medicines and Medical Products (AEMPS). Signed informed consent was obtained from all women who participated in the study. This clinical trial was registered at www.clinicaltrialsregister.eu with EudraCT number 2012-005480-28 and at www.clinicaltrials.gov with identification number NCT03196882.

See Table 1 for more information on the study design.

### 2.2. Instruments and Data Collection

#### 2.2.1. Biochemical Data

Blood samples were taken in the 12th and 36th weeks of gestation for biochemical determinations. Specifically, serum vitamin D was determined by the ADVIA Centaur VitD immunoassay method. Vitamin D levels were classified according to the cut-off point proposed by the Institute of Medicine (IOM) (2011), which defines vitamin D deficiency at levels of 25(OH)D3 < 30 nmol/L (<12 ng/mL), vitamin D insufficiency between 30–50 nmol/L (12–20 ng/mL) and vitamin D sufficiency at levels >50 nmol/L (≥30 ng/mL) [18,19]. For this study, we also defined a level <20 nmol/ (<8 ng/mL) as highly deficient. Folate and vitamin B_12_ in serum were determined using a competitive immunoassay with direct chemiluminescence technology (ADVIA Centaur, Siemens Healthineers, Madird, Spain), while serum ferritin (μg/mL) was determined using the immunochemiluminescence method.

#### 2.2.2. Psychological Data

The Bayley Scales of Infant Development (BSID-III) [20] enables the functional neurodevelopment of children aged 0 to 42 months to be assessed using four subscales: cognition, motor skills, language, and behaviour. For this study, we used the first three of these scales. The language subscale comprises two subscales to assess receptive and expressive language, while the motor subscale consists of two subscales to assess fine and gross motor skills. The results from each scale are expressed in composite scores that have an average of 100 with a standard deviation of 15, except the scores for the receptive and expressive language scales and fine and gross motor skills, which are expressed in scalar scores with a mean of 10 and a standard deviation of 3. Two trained psychologists administered the BSID-III at the visit held 40 days postpartum. After receiving training in how to jointly administer the test, they achieved a high level of agreement (98%) in the results.

The State–Trait Anxiety Inventory (STAI) [21] (Spielberger, Gorsuch, and Lushene 1988) was administered to determine the mothers’ emotional status. This self-report questionnaire is designed to measure levels of Trait Anxiety (20 items: dispositional and stable anxiety) and State Anxiety (20 items: situational and transient anxiety). For this study we used only the scores for State Anxiety. The STAI was administered at the first and third trimester visits.

The mothers completed the Parental Stress Index Short Form 4th edition (PSI-4-SF) [22]; by responding to 36 statements for measuring stress directly associated with parenting. For this study we used only the total score for Parent–Child Dysfunctional Interaction, which is related to attachment between mother and child. This questionnaire was administered during the visit held 40 days postpartum.

#### 2.2.3. Sociodemographic Data

Information about the educational level and occupational status of the participating women and their partners was recorded using the Catalan classification of occupations (CCO-2011) [23]. This information was used to calculate the families’ socioeconomic status (SES), which was estimated using the Hollingshead index [24].

#### 2.2.4. Lifestyle Habits

The Fagerström questionnaire (Fagerström_Q) [25] was used to assess smoking. The women were classified as smokers or non-smokers.

Usual Food consumption was assessed using a semiquantitative food frequency questionnaire (FFQ) validated for our population [26] at weeks 12 and 36 of pregnancy. Grams per day were calculated for the 45 food items. Diet quality was assessed with an Mediterranean Diet (rMED) score [27] based on the intake of nine components from this diet [28]. Values of 0, 1 or 2 points were assigned to each tertile of these nine components: positive for fruits, vegetables, legumes, cereals, fresh fish and olive oil, and invested for meat and dairy products. The consumption of alcoholic beverages scored 0, while non-consumption scored 2. The scores assigned to each participant ranged from 0 to 18 points (ranging from minimal to maximum degree of adherence).

#### 2.2.5. Obstetrical and Birth Data

Data on weight, length and head circumference at birth, gestational age at birth, Apgar test scores, and type of delivery were collected from the babies’ health cards. The mothers were also asked about which type of feeding they used. The gender of the babies was also recorded.

### 2.3. Statistical Analyses

Descriptive data were expressed as means and standard deviations for the quantitative variables and as percentages for the qualitative variables. Differences in infant cognitive development scales between groups of vitamin D levels were tested using the ANOVA test and Bonferroni post-hoc analysis. The n and percentage of participants in each level was also calculated. To determine how vitamin D (nmol/L) levels in the first and third trimester of pregnancy (independent variable) affect several neurodevelopmental aspects (such as cognitive, language and motor skills and outcomes), multiple linear regression models were performed. The vitamin D (nmol/L) levels were introduced using the enter method. Dummy variables were created for the various vitamin D level cut-off points. On the one hand, two dummy variables took into account the IOM cut-off points, with <30 as the reference group and <30 vs. 30–50 nmol/L and <30 vs. >50 nmol/L as the variables. On the other hand, two dummy variables took into account the highly deficient level, with <20 as the reference group and <20 vs. 20–50 nmol/L and <20 vs. >50 nmol/L as the variables. The following possible confounding variables were incorporated into the models using the stepwise method: mother’s age (years), family socioeconomic level (score), tobacco consumption (yes/no), type of feeding (formula/breastfeeding), gestational age at birth (weeks), type of delivery (eutocic/dystocic), child’s gender (0: boy/1: girl), mother–child interaction score, mother anxiety state score (first or third trimester), ferritin levels (μg/L) (first or third trimester), folates (nmol/L), B_12_ vitamin levels (ng/mL) (first or third trimester), and diet quality (first or third trimester). In the regressions to determine the effect of third trimester vitamin D levels, the models were also adjusted for first trimester vitamin D levels.

For statistical analyses we used SPSS software version 26.0 for Windows (New York, USA). Statistical significance was set at 0.05.

## 3. Results

Taking into account the IOM classification, in the first trimester we observed that 50.2% of the pregnant women had vitamin D deficiency (<30 nmol/L) (mean = 20.3; Standard Devition SD= 5.6), 30.3% had vitamin D insufficiency (30–50 nmol/L) (mean = 38.7; SD = 5.5), and 19.5% had normal vitamin D levels (>50 nmol/L) (mean = 61.6; SD 9.1). In the third trimester, 49.7% of pregnant women had vitamin D deficiency (mean = 20.3; SD 5.4), 33.2% had vitamin D insufficiency (mean = 39.1; SD = 5.8), and 17.2% had normal vitamin D levels (mean = 61.9; SD = 10.8). With regard to highly deficient levels, in the first trimester 22.8% of mothers had vitamin D levels <20 nmol/L (mean = 15.2; SD = 3.7) while in the third trimester 23.7% of mothers did (mean = 15.5; SD = 3.0). Taking into account deficient and insufficient levels along pregnancy, a 10.7% of women had normal levels at the first and third trimester of pregnancy, a 8.8% had deficient or insufficient levels at the first trimester and normal levels at the third trimester, a 6.4% of women had normal levels at first trimester and deficient or insufficient at third and a 74% of women had deficient or insufficient levels both at first and third trimesters. The mean age of the mothers was 30.6 (5.1) and about 80% of them belonged to a mid-to-high socioeconomic environment. The descriptive data showed normal anthropometric measurements at birth (mean weight = 3295.9, SD = 448.6; mean length = 49.1, SD = 2.3; and mean head circumference = 34.5, SD = 1.5) and normal Apgar scores (mean = 9.6, SD = 0.4). Moreover, 96% of the babies were born at term (see Table 2 for the descriptive results for the sample).

The psychological characteristics of the infants according to vitamin D status in the first and third trimester are shown in Table 3. Post-hoc analyses indicated that, in the first trimester, children of mothers with vitamin D deficiency (<30 nmol/L) scored significantly lower on the BSID-III cognitive scale (mean = 100.9, SD = 8.1) than children of mothers with sufficient levels of vitamin D (>50 nmol/L) (mean = 103.9, SD = 8.3). Our results also showed that children of mothers with vitamin D levels <20 nmol/L obtained significantly lower scores on all the language BSID-III scales than children of mothers with levels between 30 nmol/L and 50 nmol/L (mean = 93.5, SD = 9.3 vs. mean = 97.2, SD = 8.2 for the language composite score; mean = 10.1, SD = 2.3 vs. mean = 11.0, SD = 2.0 for the receptive language scale, and mean = 7.7, SD = 1.5 vs. mean = 8.4, SD = 1.5 for the expressive language scale). No significant differences were found in the third trimester bivariate analyses. The adjusted multiple linear regression models in Table 4 show that deficient vitamin D levels during the first trimester (<30 nmol/L) predicted lower cognitive scores. Table 5 shows that deficient vitamin D levels (<20 nmol/l and <30 nmol/L) in the first trimester were related to lower scores on language receptive and language expressive skills. This effect was especially observed for general language development when the vitamin D levels were below 20 nmol/L. The regression models also showed that highly deficient vitamin D levels (<20 nmol/L) in the third trimester negatively influenced the motor composite scores (see Table 6). Overall, the beta values did not vary when we took into account the unadjusted or adjusted models. Our results therefore show that the vitamin D effect was not reduced when we controlled for all possible confounders.

The adjusted models also highlighted variables other than low vitamin D levels that predicted the BSID-III scores. Higher gestational age at birth was associated with better performance in all areas. Moreover, higher folate levels and higher ferritin levels in the first trimester were related to better cognitive and language performance, respectively. The children of mothers with better mother–child interaction also showed higher language-related scores.

## 4. Discussion

This prospective longitudinal study of a large sample of pregnant women with no previous pathology has shown that the women’s vitamin D levels at the beginning and end of gestation had significant effects on the neurodevelopment of their children at 40 days. Low vitamin D levels were not related to low mean scores on BSID-III at this age. In all cases, our results indicated a worse performance but within the normal range on all BSID-III scales.

Regardless of other associated factors, deficient vitamin D levels in the first trimester of pregnancy predicted worse performance in cognitive and language skills. Language performance especially was significantly lower when vitamin D levels were highly deficient (<20 nmol/L). During the third trimester, this highly deficient vitamin D level also affected motor development.

With regard to cognitive development, after controlling for numerous possible confounders our results showed that, together with lower gestational ages at birth and lower levels of folates, deficient vitamin D levels in the first trimester (<30 nmol/L) were associated with worse cognitive outcomes than normal levels. These results are in agreement with the findings of Morales et al. [12], who observed that children of mothers with vitamin D levels <50 nmol/L at 13.5 ± 2.1 weeks of gestation had lower mental index scores at 14 months of age than children of mothers whose levels were above 75 nmol/L. The relationship between vitamin D during gestation and calcium and bones is widely known. However, it is only in recent decades that the influence of prenatal vitamin D levels on brain development has been understood. The early stages of gestation (first and second trimesters) are a critical period in the development of the foetal nervous system due to the beginning of neurogenesis and the myelination process [29] and, as we know, foetuses are utterly dependent on their mothers’ vitamin D status. This could be why low levels of this vitamin during this period may be closely related to the children’s neurodevelopment [30,31] and why this effect on cognitive and language skills is not observed when the vitamin D deficiency occurs at the end of gestation. In our study we assessed the effect on neurodevelopment at 40 days, as other authors have [13,15]. However, more follow-up studies are needed on the long-term effects (i.e., during childhood and adolescence) of low levels of vitamin D during the first trimester. In this regard, previous research has found no association between deficient vitamin D levels during the third trimester of pregnancy and children’s cognitive function, including IQ (Intelligence Quotient) measured during childhood and adolescence [14,32]. In those studies, the analyses were also adjusted for several possible parental and child confounding factors. Specifically, in agreement with our results, in their study of a sample of Indian children aged 9–10 and 13–14, Veena et al. [32] found no association between vitamin D levels measured in the third trimester (around 30 weeks of gestation) and children’s cognitive function. Consistent with these findings, two other European studies found that vitamin D levels measured in the third trimester were also unrelated to children’s IQ or academic performance [33,34]. As Veena et al. [32] suggested, there may be a critical period for neurodevelopment, the first half of the pregnancy, during which vitamin D may be more needed. Unlike these studies, we were able to compare the effects of both measurements since we assessed vitamin D at two gestational periods.

In addition to cognitive development, vitamin D levels at the beginning of gestation were also related to language development, which again indicates that this is possibly the most critical period of pregnancy for foetal and child development. Our results showed that, like deficient vitamin D levels during the first trimester of pregnancy, lower gestational age, poor mother–child interaction, and low ferritin levels were related to worse performance in language skills. More specifically, the most deficient levels of vitamin D (<20 nmol/L) were associated with poorer performance on all language scales. These results are similar to those found by Janbek et al. [5], who concluded that an association exists between hypovitaminosis D during pregnancy and worse performance in expressive language that may even persist into adolescence. Whitehouse et al. [35] observed that the offspring of Australian mothers with low vitamin D levels at the beginning of the second trimester (whether these levels were already low in the first trimester was unknown) were almost twice as likely to have more severe language impairment at five and ten years of age than those of mothers with high vitamin D levels (Odds Ratio (OR) = 1.97; 95%Confidence Interval (CI) = 1.00–3.93). In contrast, after controlling results for several environmental factors, a study conducted in the United States found that higher gestational 25(OH)D3 status in the second trimester was significantly associated with higher scaled scores for receptive language, but not for expressive language, when the children were two years old [36]. In general, studies support the effect of 25(OH)D3 on early language development during the first stages of pregnancy. In our case this was observed as early as 40 days postpartum. Since brain development begins with neural plate formation only 12 days after conception but continues throughout early adulthood, we are currently following up the present sample to check whether the effects of vitamin D deficiencies during pregnancy on offspring neurodevelopment are maintained at four years of age. Our results also showed that the vitamin D effect on language performance is not reduced after controlling for numerous possible confounding factors. Both our results and those of previous studies show that language development is closely related to mother–child attachment. In this respect, [37] suggested that maternal sensitivity and maternal language in infancy make important contributions to a child’s language development. Other studies have suggested that iron deficiency in foetal life is associated with poor neurodevelopmental outcomes, though results from epidemiological studies are still inconsistent [38,39]. In line with the relationship observed between ferritin levels and language scores, the results of a study of 331 pregnant women conducted by Berglund et al. [40] indicated that maternal iron deficiency at delivery was associated with lower receptive, expressive and composite BSID-III language scores in offspring at 18 months of life. It is important, therefore, to promote adequate nutrition at the beginning of pregnancy in order to optimize neurodevelopmental outcomes. Georgieff et al. [41], for their part, concluded that optimizing nutrition during foetal and early postnatal life is an opportunity to improve neurodevelopment and brain function across the lifespan.

With regard to psychomotor development, no results were observed with the levels established by the IOM. However, the regression models showed that highly deficient levels of vitamin D (<20 nmol/L) in the third trimester predicted worse psychomotor performance 40 days postpartum. Similarly, after adjusting for several possible confounders (maternal age, body mass index, ethnicity, child’s gender, breastfeeding, tobacco consumption in the first trimester, parity, oily fish intake, socioeconomic factors, weeks of gestation, and the season in which the sample was collected), Darling et al. [14] observed—in a study conducted in southwest England with a total of 7065 mother–child pairs (median gestational week of vitamin D measurement = 29.6 weeks: 26.1% measured in the first trimester, 11.8% measured in the second, and 62.1% measured in the third)—that children of vitamin D deficient mothers (<50 nmol/l) were more likely to obtain scores in the lowest quartile for gross-motor and fine-motor development at 30 months (OR = 1.20; 95%CI = 1.03–1.40; and OR = 1.23; 95%CI = 1.05–1.44, respectively). Since, as in our study, Darling et al. [14] assessed vitamin D during the third trimester in over 60% of the sample, data suggest that vitamin D deficit in late gestation may influence motor skill outcomes. Dhamayanti et al. [11] recently conducted a cohort study in Indonesia in which maternal serum 25(OH)D3 was measured at 10–14 weeks of pregnancy. Unlike in our study, they found that vitamin D deficiency in the first trimester was related to lower scores in gross motor function at the age of three months. Dhamayanti et al.’s findings [11] were adjusted for a lower number of confounding variables than ours but the differences may also be explained by the fact that Indonesian mothers may have different nutritional or psychosocial risk factors than those in our sample. Motor development is closely related to the myelination process and vitamin D is known to act on myelination. Neural pathways involved in the acquisition of psychomotor function may therefore be more vulnerable to this deficiency or insufficiency throughout pregnancy [42,43,44,45]. It is also interesting to note that myelination occurs mainly in the third trimester and continues postnatally.

Our results repeatedly show that higher gestational age is clearly related to better neurodevelopmental outcomes. More weeks of gestation at birth are also known to be associated with better maturity levels of the nervous system [46].

The main limitation of our study is that, despite our efforts, participation at the visits held 40 days postpartum was lower than expected. This may be because, like pregnancy follow-up visits, these visits were not compulsory. At the same time, a baby’s arrival inevitably involves a certain amount of stress as well as family planning difficulties. On the other hand, this research provides current evidence on the impact of vitamin D levels during pregnancy on early neurodevelopment by analysing a sample of mother–infant pairs from a Mediterranean environment while rigorously adjusting for a wide range of confounding factors. Unlike numerous other studies, we also adjusted for the mothers’ psychological state during pregnancy and their attachment to their offspring, as well as several other variables related to maternal prenatal diet, family socioeconomic level, and obstetrical and neonatal factors. Moreover, unlike other studies that used a composite score to represent overall development [32,47], we explored how the vitamin D levels affected three specific neurodevelopment subscales. In addition, we studied the impact of the deficient vitamin D levels as defined by the IOM and also considered highly deficient levels (<20 nmol/L) in two periods (beginning and end of gestation). Note also that neurodevelopment assessment was conducted early (i.e., 40 days after birth), when other environmental factors could hardly have had any influence.

The rates of pregnant women with suboptimal vitamin D levels are very high worldwide. In our sample this prevalence reached 50% in the first trimester (unpublished results) and 49.7% in the third (<30 nmol/L). In the first trimester roughly 22.8% of women had vitamin D deficiency below 20 nmol/L. In the third trimester this figure was 23.7%. In general, our results show that the offspring of mothers with normal prenatal vitamin D levels significantly improved their performance in cognitive, language, and motor skills. Hypovitaminosis D during pregnancy could therefore be an important public health concern with dire consequences for gestational course, the mother’s and child’s health, and the child’s development throughout their lifespan. Measuring vitamin D early in pregnancy could present an excellent opportunity to identify at-risk pregnancies in a timely fashion, thus providing ample time to monitor such pregnancies and offering early prophylaxis [48]. However, our results should be interpreted with an element of caution due to heterogeneity between studies. As de-Regil et al. [49] suggested, evidence on whether vitamin D supplements should be provided to women as a component of routine prenatal care aimed at improving maternal and child outcomes is still unclear. More studies are needed both to clarify the precise role of vitamin D and to produce specific nutritional recommendations for pregnant women to follow to ensure the proper course of their pregnancy, optimal foetal brain development, and optimal neurodevelopment of their child throughout their lifespan.

## Figures and Tables

**Table 1:** Study phases.

RECRUITMENT ≤ 12 Weeks	1st Trimester Visit 12th Week	3rd Trimester Visit 36th Week	Postnatal Visit 40 Days Postpartum
	N = 793 pregnant women		N = 422 mother–infant
			pairs
	Sociodemographic data: - Mother’s age - Mother’s and father’s occupational status - Mother’s and father’s educational level		Obstetrical and birth data: - Type of delivery - Gestational age - Birth weight - Birth height - Cranial perimeter
	Lifestyle habits: - Smoking (Fagerström-Q) - Diet quality (rMED)	Lifestyle habits: - Diet quality (rMED)	- Type of feeding - Neurodevelopmental skills (BSID-III) - Mother–child interaction (PSI)
	Psychological state: - State–Trait Anxiety Inventory (STAI)	Psychological state: - State–Trait Anxiety Inventory (STAI)	
	Blood test: - Vitamin D - Vitamin B_12_ - Ferritin - Folates	Blood test: - Vitamin D - Vitamin B_12_ - Ferritin	

rMED: Mediterranean diet score; BSID-III: Bayley Scales for Infant Development; PSI: Parenting Stress Index.

**Table 2:** Mother and offspring descriptive data: sociodemographic data, health habits, nutrition and psychological aspects.

Mothers		Offspring	
Mother’s age, mean (SD)	30.6 (5.1)	Gender (%)	
Socioeconomic level (%)		Boys	49.1
Low	15.6	Girls	50.9
Mid	68.3	Birth weight, mean (SD)	3295.9 (448.6)
High	16.0	Birth length, mean (SD)	49.1 (2.3)
STAI—State anxiety, mean (SD)		Birth head circumference, mean (SD)	34.5 (1.5)
First trimester	17.9 (8.8)	Apgar, mean (SD)	9.6 (0.4)
Third trimester	19.3 (8.7)		
rMED—Diet quality, mean (SD)		BSID-III, mean (SD)	
First trimester	9.5 (2.6)	Cognitive scale	101.9 (8.8)
Third trimester	9.9 (2.6)	Language scale	96.2 (8.4)
Tobacco consumption during pregnancy (%)		Receptive	10.6 (2.1)
Yes	15.3	Expressive	8.1 (1.6)
No	84.7	Motor scale	107.9 (11.5)
Gestational age at birth, mean (SD)	39.7 (1.4)	Fine	11.5 (1.9)
Type of delivery (%)		Gross	11.1 (2.3)
Eutocic	66.7		
Dystocic	33.3	PSI, Mother–child interaction mean (SD)	50.7 (7.9)
Preterm birth (%)			
Yes	3.8		
No	96.2		
Type of feeding (%)			
Formula	18.6		
Breasfeeding	81.4		

STAI: State–Trait Anxiety Inventory; rMED: Mediterranean diet score; BSID-III: Bayley Scales of Infant Development-III; PSI: Parenting Stress Index.

**Table 3:** Bivariate analyses between vitamin D levels during pregnancy per trimester and neurodevelopmental offspring data.

									ANOVA 1		ANOVA 2	
	<20 nmol/L ^a^		<30 nmol/L ^b^		30–50 nmol/L ^c^		>50 nmol/L ^d^		p	Bonferroni	p	Bonferroni
First trimester of pregnancy vitamin D levels, n(%)	181 (22.8)		398 (50.2)		240 (30.3)		155 (19.5)					
BSID-III (scores)												
Cognitive scale	101.1	(7.6)	100.9	(8.1)	101.8	(9.8)	103.9	(8.3)	0.029	0.024 bd	0.068	
Language scale	93.5	(9.3)	95.2	(8.4)	97.2	(8.2)	96.9	(8.5)	0.079		0.019	0.015 ac
Receptive	10.1	(2.3)	10.4	(2.1)	11.0	(2.0)	10.6	(2.2)	0.056		0.030	0.018 ac
Expressive	7.7	(1.5)	7.9	(1.5)	8.1	(1.7)	8.4	(1.5)	0.095		0.049	0.031 ad
Motor scale	107.3	(10.8)	107.6	(11.0)	108.1	(12.6)	108.0	(10.8)	0.904		0.963	
Fine	11.3	(1.8)	11.4	(1.9)	11.7	(2.0)	11.4	(1.9)	0.404		0.540	
Gross	11.1	(2.6)	11.1	(2.4)	11.2	(2.0)	11.2	(2.5)	0.827		0.919	
Third trimester of pregnancy vitamin D levels, n(%)	188 (23.7)		394 (49.7)		263 (33.2)		136 (17.2)					
BSID-III (scores)												
Cognitive scale	101.7	(7.2)	101.6	(8.4)	101.7	(9.5)	102.8	(8.4)	0.577		0.776	
Language scale	94.9	(8.8)	95.8	(8.2)	96.5	(8.5)	96.7	(8.8)	0.611		0.470	
Receptive	10.2	(2.2)	10.5	(2.1)	10,8	(2.0)	10.6	(2.3)	0.537		0.258	
Expressive	8.0	(1.6)	8.0	(1.5)	8.0	(1.7)	8.2	(1.6)	0.637		0.815	
Motor scale	105.8	(14.4)	107.3	(12.1)	108.1	(10.8)	108.9	(11.4)	0.566		0.341	
Fine	11.2	(1.9)	11.4	(2.0)	11.6	(1.9)	11.5	(1.9)	0.592		0.407	
Gross	11.1	(2.3)	11.1	(2.2)	11.0	(2.3)	11.4	(2.5)	0.502		0.703	

BSID-III: Bayley Scales of Infant Development-III. ANOVA 1: comparisons between <30 nmol/L, 30–50 nmol/L, >50 nmol/L. ANOVA 2: comparisons between <20 nmol/L, 20–30 nmol/L, 30–50 nmol/L, >50 nmol/L; bd; ac; ad: Differences between groups.

**Table 4:** Multiple linear regression models to explore the relationship between vitamin D during pregnancy (first and third trimesters) and the Bayle Scales of Infant Development-III (BSID-III) cognitive scale scores at 40 days postpartum.

CRITERIA: Cognitive Scales					
IOM Levels	First Trimester of Pregnancy	Third Trimester of Pregnancy	<20 nmol/L Levels	First Trimester of Pregnancy	Third Trimester of Pregnancy
Unadjusted Models	Beta *p*	Beta *p*		Beta *p*	Beta *p*
Vitamin D (<30 vs. 30–50 nmol/L)	0.045 0.426	−0.008 0.918	Vitamin D (<20 vs. 20–50 nmol/L)	0.001 0.990	0.022 0.801
Vitamin D (<30 vs. >50 nmol/L)	0.150 0.008	−0.015 0.836	Vitamin D (<20 vs. >50 nmol/L)	0.134 0.050	0.002 0.979
	*R*^2^_c_*100 = 1.4%	*R*^2^_c_*100 = −0.9%		*R*^2^_c_*100 = 1.2%	*R*^2^_c_*100 = −0.9%
	F_2.364_ = 3.627	F_2.222_ = 0.022		F_2.364_ = 3.304	F_2.222_ = 0.048
	*p* = 0.028	*p* = 0.978		*p* = 0.038	*p* = 0.953
**Adjusted models**					
Vitamin D (<30 vs. 30–50 nmol/L)	0.051 0.354	−0.017 0.809	Vitamin D (<20 vs. 20–50 nmol/L)	−0.001 0.986	0.057 0.508
Vitamin D (<30 vs. >50 nmol/L)	0.141 0.011	−0.001 0.985	Vitamin D (<20 vs. >50 nmol/L)	0.121 0.074	0.043 0.619
Gestational age	0.175 0.001	0.258 0.001	Gestational age	0.174 0.001	0.262 0.001
Folate	0.103 0.046		Folate	0.102 0.050	
	*R*^2^_c_*100 = 4.6%	*R*^2^_c_*100 = 5.3%		*R*^2^_c_*100 = 4.3%	*R*^2^_c_*100 = 5.5%
	F_4.364_ = 5.353	F_3.222_ = 5.179		F_4.364_ = 5.126	F_3.222_ = 5.316
	*p* = 0.001	*p* = 0.002		*p* = 0.001	*p* = 0.001

Models adjusted for the following variables: vitamin D levels (first or third trimester); mother’s age (years), family socioeconomic level (score), tobacco consumption (yes/no), type of feeding (formula/breastfeeding), gestational age at birth (weeks), type of delivery (eutocic/dystocic), child’s gender (0: boy/1: girl), mother–child interaction score, mother anxiety state score (first or third trimester), ferritin levels (μg/mL) (first or third trimester), folates (μg/mL), B_12_ vitamin levels (μg/mL) (first or third trimester), and diet quality (first or third trimester). The models for the third trimester were adjusted for the first trimester vitamin D levels. IOM: Institute Of Medicine. *R*^2^_c_*100: Corrected *R* square multiplied by 100.

**Table 5:** Multiple linear regression models to explore the relationship between vitamin D during pregnancy (first and third trimester) and the Bayle Scales of Infant Development-III (BSID-III) language scores at 40 days postpartum.

CRITERIA: Language Receptive Scale					
IOM Levels	First Trimester of Pregnancy	Third Trimester of Pregnancy	<20 nmol/L Levels	First Trimester of Pregnancy	Third Trimester of Pregnancy
Unadjusted models	Beta *p*	Beta *p*		Beta *p*	Beta *p*
Vitamin D (<30 vs. 30–50 nmol/L)	0.120 0.034	0.044 0.546	Vitamin D (<20 vs. 20–50 nmol/L)	0.164 0.017	0.144 0.104
Vitamin D (<30 vs. >50 nmol/L)	0.038 0.498	−0.003 0.969	Vitamin D (<20 vs. >50 nmol/L)	0.100 0.145	0.073 0.411
	*R*^2^_c_*100 = 0.7%	*R*^2^_c_*100 = −0.7%		*R*^2^_c_*100 = 1.0%	*R*^2^_c_*100 = 2.0%
	F_2.364_ = 2.272	F_2.222_ = 0.231		F_2.364_ = 2.861	F_2.222_ = 1.378
	*p* = 0.105	*p* = 0.794		*p* = 0.058	*p* = 0.254
**Adjusted models**					
Vitamin D (<30 vs. 30–50 nmol/L)	0.122 0.029		Vitamin D (<20 vs. 20–50 nmol/L)	0.166 0.015	0.163 0.065
Vitamin D (<30 vs. >50 nmol/L)	0.030 0.595		Vitamin D (<20 vs. >50 nmol/L)	0.092 0.176	0.095 0.280
Gestational age	0.136 0.009		Gestational age	0.136 0.009	0.146 0.030
	*R*^2^_c_*100 = 2.3%			*R*^2^_c_*100 = 2.6%	*R*^2^_c_*100 = 2.0%
	F_3.364_ = 3.822			F_3.364_ = 4.218	F_3.222_ = 2.522
	*p* = 0.010			*p* = 0.006	*p* = 0.059
**CRITERIA: Language expressive scale score**					
IOM levels	First trimester of pregnancy	Third trimester of pregnancy	<20 nmol/L levels	First trimester of pregnancy	Third trimester of pregnancy
Unadjusted models	Beta *p*	Beta *p*		Beta *p*	Beta *p*
Vitamin D (<30 vs. 30–50 nmol/L)	0.021 0.706	0.054 0.465	Vitamin D (<20 vs. 20–50 nmol/L)	0.103 0.113	0.079 0.376
Vitamin D (<30 vs. >50 nmol/L)	0.127 0.025	0.031 0.677	Vitamin D (<20 vs. >50 nmol/L)	0.186 0.007	0.060 0.498
	*R*^2^_c_*100 = 0.9%	*R*^2^_c_*100 = −0.7%		*R*^2^_c_*100 = 1.5%	*R*^2^_c_*100 = 7.6%
	F_2.364_ = 2.659	F_2.222_ = 0.277		F_2.364_ = 3.738	F_2.222_ = 0.403
	*p* = 0.071	*p* = 0.759		*p* = 0.025	*p* = 0.669
**Adjusted models**					
Vitamin D (<30 vs. 30–50 nmol/L)	0.011 0.847	0.024 0.822	Vitamin D (<20 vs. 20–50 nmol/L)	0.097 0.150	0.069 0.422
Vitamin D (<30 vs. >50 nmol/L)	0.116 0.036	0.009 0.811	Vitamin D (<20 vs. >50 nmol/L)	0.175 0.009	0.055 0.522
PSI Mother–child interaction	0.191 0.001	0.215 0.976	PSI Mother–child interaction	0.190 0.001	0.203 0.002
Ferritin	0.108 0.037	0.158 0.973	Ferritin	0.106 0.039	0.172 0.010
Tobacco use		−0.141 0.972	Tobacco use		−0.150 0.023
	*R*^2^_c_*100 = 4.8%	*R*^2^_c_*100 = 6.1%	Gestational age		0.134 0.043
	F_4.364_ = 5.566	F_5.222_ = 3.901		*R*^2^_c_*100 = 5.3%	*R*^2^_c_*100 = 7.6%
	*p* = 0.001	*p* = 0.002		F_4.364_ = 6.109	F_6.222_ = 4.033
				*p* = 0.001	*p* = 0.001

Models adjusted for the following variables: vitamin D levels (first or third trimester); mother’s age (years), family socioeconomic level (score), tobacco consumption (yes/no), type of feeding (formula/breastfeeding), gestational age at birth (weeks), type of delivery (eutocic/dystocic), child’s gender (0: boy/1: girl), mother–child interaction score, mother anxiety state score (first or third trimester), ferritin levels (μg/mL) (first or third trimester), folates (μg/mL), B_12_ vitamin levels (μg/mL) (first or third trimester), and diet quality (first or third trimester). The models for the third trimester were adjusted for first trimester vitamin D levels.

**Table 6:** Multiple linear regression models to explore the relationship between vitamin D during pregnancy (first and third trimester) and the Bayle Scales of Infant Development-III (BSID-III) motor scores at 40 days postpartum.

CRITERIA: Fine motor score					
IOM Levels	First Trimester of Pregnancy	Third Trimester of Pregnancy	<20 nmol/L Levels	First Trimester of Pregnancy	Third Trimester of Pregnancy
Unadjusted Models	Beta *p*	Beta *p*		Beta *p*	Beta *p*
Vitamin D (<30 vs. 30–50 nmol/L)	0.041 0.001	−0.012 0.869	Vitamin D (<20 vs. 20–50 nmol/L)	0.055 0.428	0.126 0.155
Vitamin D (<30 vs. >50 nmol/L)	−0.016 0.466	0.013 0.860	Vitamin D (<20 vs. >50 nmol/L)	0.004 0.950	0.100 0.259
	*R*^2^_c_*100 = −0.3%	*R*^2^_c_*100 = −0.9%		*R*^2^_c_*100 = −0.3%	*R*^2^_c_*100 = 0%
	F_2.364_ = 0.444	F_2.222_ = 0.049		F_2.364_ = 0.493	F_2.222_ = 1.054
	*p* = 0.642	*p* = 0.952		*p* = 0.611	*p* = 0.350
**Adjusted models**					
Vitamin D (<30 vs. 30–50 nmol/L)	0.061 0.276	−0.034 0.641	Vitamin D (<20 vs. 20–50 nmol/L)	0.048 0.481	
Vitamin D (<30 vs. >50 nmol/L)	−0.005 0.924	−0.012 0.869	Vitamin D (<20 vs. >50 nmol/L)	0.006 0.927	
rMED first trimester	0.152 0.004		rMED first trimster	0.138 0.009	
Gestational age	0.113 0.029		Gestational age	0.117 0.025	
Tobacco use	0.104 0.045			*R*^2^_c_*100 = 2.3%	
Gender		0.158 0.020		F_4.364_ = 3.124	
Socioeconomic level		−0.145 0.032		p = 0.015	
	*R*^2^_c_*100 = 3.2%	*R*^2^_c_*100 = 2.2%			
	F_5.364_ = 3.436	F_4.222_ = 2.263			
	*p* = 0.005	*p* = 0.063			
**CRITERIA: Gross motor score**					
IOM levels	First trimester of pregnancy	Third trimester of pregnancy	<20 nmol/L levels		
Unadjusted models	Beta *p*	Beta *p*			
Vitamin D (<30 vs. 30–50 nmol/L)	0.014 0.802	−0.029 0.695	Vitamin D (<20 vs. 20–50 nmol/L)	0.015 0.827	0.129 0.144
Vitamin D (<30 vs. >50 nmol/L)	−0.015 0.788	0.056 0.448	Vitamin D (<20 vs. >50 nmol/L)	−0.011 0.877	0.151 0.087
	*R*^2^_c_*100 = −0.5%	*R*^2^_c_*100 = −0.4%		*R*^2^_c_*100 = −0.5%	*R*^2^_c_*100 = 0.5%
	F_2.364_ = 0.107	F_2.222_ = 0.582		F_2.364_ = 0.100	F_2.222_ = 1.581
	*p* = 0.898	*p* = 0.560		*p* = 0.905	*p* = 0.208
**Adjusted models**					
Vitamin D (<30 vs. 30–50 nmol/L)	0.021 0.711		Vitamin D (<20 vs. 20–50 nmol/L)	0.013 0.848	
Vitamin D (<30 vs. >50 nmol/L)	−0.027 0.633		Vitamin D (<20 vs. >50 nmol/L)	−0.026 0.705	
Gestational age	0.130 0.013		Gestational age	0.130 0.013	
Tobacco use	0.112 0.033		Tobacco use	0.111 0.034	
	*R*^2^_c_*100 = 2.0%			*R*^2^_c_*100 = 2.0%	
	F_4.364_ = 2.903			F_4.364_ = 2.877	
	*p* = 0.022			*p* = 0.023	

Models adjusted for the following variables: vitamin D levels (first or third trimester); mother’s age (years), family socioeconomic level (score), tobacco consumption (yes/no), type of feeding (formula/breastfeeding), gestational age at birth (weeks), type of delivery (eutocic/dystocic), child’s gender (0: boy/1: girl), mother–child interaction score, mother anxiety state score (first or third trimester), ferritin levels (μg/mL) (first or third trimester), folates (μg/mL), B_12_ vitamin levels (μg/mL) (first or third trimester), and diet quality (first or third trimester). The models for the third trimester were adjusted for first trimester vitamin D levels.
